# Exploring community needs in combating aedes mosquitoes and dengue fever: a study with urban community in the recurrent hotspot area

**DOI:** 10.1186/s12889-024-18965-1

**Published:** 2024-06-20

**Authors:** Nurul Adilah Samsudin, Hidayatulfathi Othman, Ching Sin Siau, Zul-‘Izzat Ikhwan Zaini

**Affiliations:** 1 Centre for Toxicology and Health Risk Studies (CORE), Faculty of Health Sciences, Universiti Kebangsaan Malaysia, Kuala Lumpur, 53000 Malaysia; 2 Centre for Community Health Studies (ReaCH), Faculty of Health Sciences, Universiti Kebangsaan Malaysia, Kuala Lumpur, 53000 Malaysia; 3 Department of Basic Science, Faculty of Health Sciences, Universiti Teknologi Mara (UiTM), Cawangan Pulau Pinang, Kampus Bertam, Kepala Batas, Penang, 13200 Malaysia

**Keywords:** Community-based participatory research (CBPR), Aedes mosquito, Dengue prevention and control, Community needs, Hulu Langat

## Abstract

**Background:**

Aedes mosquitoes are the main vector of dengue infection, a global health threat affecting millions of people annually. Conventional prevention and control methods against dengue outbreaks have only achieved marginal success. Recognizing the complex issue at hand, a multilevel participatory approach is crucial. Thus, alternative strategies that involve community engagement are increasingly being considered and attempted. While community-based vector control programs have been conducted, sustaining behavioral changes among the population remains a challenge. This study aimed to identify the specific community needs in combating Aedes mosquitoes and dengue fever as a basis to guide the development of community-driven initiatives and foster a deeper sense of ownership in the fight against dengue.

**Methods:**

Between 1 August 2022 and 30 November 2022, we conducted a study in Hulu Langat district, Selangor, using a mixed-method design. All participants consented to the study, which comprised 27 participants (FGDs) and 15 participants (IDIs). The IDIs included two participants with a history of dengue fever, one community leader, one faith leader, seven local authorities, and four district health officers. Semi-structured interviews and discussions were performed among stakeholders and community members recruited via purposive and snowball sampling techniques. All interviews were audio-recorded before being analyzed using reflexive thematic analysis.

**Results:**

These results derived from qualitative data explored the perspectives and needs of communities in combating Aedes mosquitoes and dengue fever. Interviews were conducted with various stakeholders, including community members, leaders, and health officers. The study identified the necessity of decisive actions by authorities to address the impact of the dengue epidemic, the importance of community engagement through partnerships and participatory approaches, the potential benefits of incentives and rewards to enhance community participation, and the need for sustained community engagement and education, especially via the involvement of young people in prevention efforts. These findings provide valuable insights into the design of effective strategies against Aedes mosquitoes and dengue fever.

**Conclusions:**

In short, there is an urgent need for a comprehensive approach involving multiple stakeholders in the fight against Aedes mosquitoes and dengue fever. The approach should incorporate efforts to raise awareness, provide practical resources, and foster community responsibility. The active involvement of teenagers as volunteers can contribute to long-term prevention efforts. Collaboration, resource allocation, and community engagement are crucial for effective dengue control and a healthier environment.

**Supplementary Information:**

The online version contains supplementary material available at 10.1186/s12889-024-18965-1.

## Background

Aedes mosquitoes pose a significant public health threat worldwide because they are the main vectors of dengue fever, a viral illness affecting millions annually [1, 2]. In recent decades, the global incidence rate of dengue fever has surged, with reported cases to the World Health Organization (WHO) increasing from 505,430 in 2000 to 5.2 million in 2019, reflecting a significant rise in new cases within the global population over the same period. Currently, approximately half of the world’s population is at risk of dengue, with an estimated 100–400 million infections occurring annually [3]. Conventional approaches such as the use of larvicides and insecticides have yielded only limited success in mosquito control and curbing the transmission of dengue fever [4, 5]. Therefore, alternative strategies that go beyond environmental interventions are warranted, included a higher level of community engagement [4, 6].

In the broader context, our approach aligns with the principles of Integrated Vector Management (IVM). IVM is a rational decision-making process aimed at optimizing the use of resources for vector control, with the goal of making vector control more efficient, cost-effective, ecologically sound, and sustainable [7]. By integrating IVM principles into community-driven initiatives, we can use local evidence, implement interventions as needed, and promote collaboration across sectors, including exploring all collaboration options within and between public and private sectors, decentralizing planning and decision-making, and improving communication among policy-makers. Additionally, local community leaders are essential for ensuring the continuity and effectiveness of behavior change efforts in Aedes mosquito control, as they mobilize communities, disseminate preventive information, and foster a collective sense of responsibility. The key elements of IVM, encompassing social mobilization, environmental management, epidemiological and entomological surveillance, and chemical and biological control, resonate with the comprehensive and inclusive approach central to our study [7].

Recognizing the complex interplay between mosquitoes, the environment, and human behaviors, a community-based participatory approach is critical in ensuring effective prevention and control of dengue fever [4, 8]. Furthermore, it is vital to engage communities from an early stage to ensure that culturally appropriate and sustainable interventions are designed and implemented [9, 10]. In the past two decades, community-based vector control programs have played a significant role in educating communities about the importance of dengue prevention [11, 12]. However, traditional approaches often overlook the specific needs of the communities affected by dengue fever, thus hindering prevention and control efforts [4, 6]. As such, a participatory approach should be prioritized, as it enables communities to be involved in designing and implementing suitable strategies tailored to their needs [10, 13].

Many countries have recognized the value of community participation, social mobilization, and behavior change communication in disease prevention, leading to the incorporation of these aspects in the relevant national guidelines [13, 14]. However, initiating and sustaining behavioral change remains a main obstacle. Without behavioral change, it is challenging to effectively implement dengue prevention and control activities [15, 16]. While guidelines and programs have been formulated, it remains a complex task to instigate behavioral changes within communities to sustain the prevention and control programs aimed at preventing dengue outbreaks [4, 17]. To overcome this, it is crucial to address the community’s specific needs so that the relevant interventions can be tailored to the local context. Active involvement of the local community in program planning and implementation is the first step toward successful dengue control [12, 16].

In this study, we set out to better understand the community’s needs in battling Aedes mosquitoes and dengue fever. The underpinning theory that guided the study conceptualization was the ideation model that focuses on the cognitive, emotional, and social factors that shape the intention of behaviors [18]. This model can be used to guide the research process and ensure the relevance and value of the collected data. A published study [19] showed that this model provided vital insights into how different factors interact and influence decisions. However, to our knowledge, no study has implemented this theory in the context of dengue fever. Therefore, we aimed to apply this model to obtain an in-depth understanding of the community’s specific needs in combating Aedes mosquitoes and dengue fever.

This study conducted an assessment of challenges and gaps within existing mosquito control and disease prevention strategies, taking into account the perspectives of both the community and stakeholders. Through an exploration of challenges experienced by community members and the collection of information regarding their opinions, knowledge, and practices related to the mosquito control and dengue prevention, our aim was to generate the necessary evidence for informing the development of community-driven initiatives. Active community participation in the fight against Aedes mosquitoes can empower individuals and instill a sense of ownership, ultimately leading to more effective and sustainable dengue prevention and control techniques [12, 15].

## Methods

### Study design

This study, conducted as part of a mixed-method design between 1 August 2022 and 30 November 2022, primarily emphasizes qualitative (QUAL) data. During this period, in-depth semi-structured interviews were conducted with gatekeepers and members of the local community. While the study also collected quantitative (QUAN) data through questionnaires assessing knowledge, attitude, and practices among local community members, the primary focus was on the qualitative component. The study design was guided by the Community-based participatory research (CBPR) methodology, recognized as crucial for a comprehensive exploration of the nuances within the community’s experiences, perceptions, and local knowledge related to Aedes mosquitoes and dengue fever prevention [20, 21].

While quantitative studies offer valuable insights into the prevalence and extent of critical health issues, the qualitative strand of this research aimed to illuminate the underlying reasons behind people’s thoughts and emotions, influencing their responses to health situations. This qualitative exploration, particularly essential in deciphering patterns in recurrent hotspot areas, provided a nuanced understanding that quantitative data alone might not capture fully [22].

All participants gave verbal and written consent, including the use of audio and video recording. The interviews were conducted at the participant’s homes or their preferred locations to ensure their privacy and comfort.

### Study location

This study was conducted in the Hulu Langat district of Selangor, the fifth largest district by area (82,994 hectares) out of the nine districts in Selangor [23]. Based on the 2020 population census, Hulu Langat district recorded the third highest population (1,400,461) in the country, after the Petaling and Johor Bahru districts.

There are two municipal councils under the Hulu Langat district, namely, the Kajang Municipal Council (MPKj) and Ampang Jaya Municipal Council (MPAJ). MPKj covers an administrative area of ​​78,761 hectares, encompassing Mukim Kajang, Cheras, Semenyih, Beranang, Hulu Langat, and Hulu Semenyih while MPAJ is responsible for Mukim Ampang [24]. This study was conducted in one of the housing estates in the Hulu Langat district which has been classified as one of the recurrent dengue hotspots in Selangor [25].

### Participants and recruitment

Purposive sampling was used to select local health officers (LO) and district health officers (DO) for individual in-depth interviews (IDI). Those with over two years of experience in the dengue vector control unit were considered. The interviews covered a range of dengue-related topics, including community lifestyles, main mosquito vectors, preferred breeding sites, community modules or policy applications, and dengue control activities.

The selection of the locality for interviews with community leaders was based on consensus during a needs assessment session with local authorities. The targeted population consisted of same local residential households located in recurrent dengue hotspot area in Hulu Langat, Selangor. Community leaders, often presidents of the “*Persatuan Penduduk*” or Residents’ Association were approached. The leaders suggested suitable individuals for interviews, including other community leaders, faith leaders, and targeted individuals (with dengue experience). The recruitment for IDIs involved both purposive and snowballing techniques.

For focus group discussions (FGDs), convenience sampling was employed to select target participants in the research area through word-of-mouth referrals and personal contacts of the research team. The inclusion criteria were: (i) aged 18 years or over, (ii) willing and able to provide written informed consent, (iii) Malaysian citizenship, (iv) able to speak Malay, and (v) they had been staying in their existing housing unit for the past 2 years (2020–2021). Subsequently, the FGD participants were also asked to recommend their friends or acquaintances who met the inclusion criteria to the researchers via the snowball sampling method.

### Ethical approval

The study was approved by the Medical Ethics Committee of the National University of Malaysia (UKM PPI/111/n8/JEP-2022-503). This was supported by Medical Research and Ethics Committee (MREC); NMRR ID-22-00314-FWY (IIR), under Ministry of Health Malaysia (MOH) and the Selangor State Department; JKNS/KA/Q-712/04 − 01 Jld 19 (25), as the study was conducted in the Health District of Hulu Langat and Selangor State Health Department. Participation in this study was voluntary and all participants provided written informed consent. All information was collected anonymously, and the outcomes were used for research purposes only.

### Data Collection

**Fig. 1:**
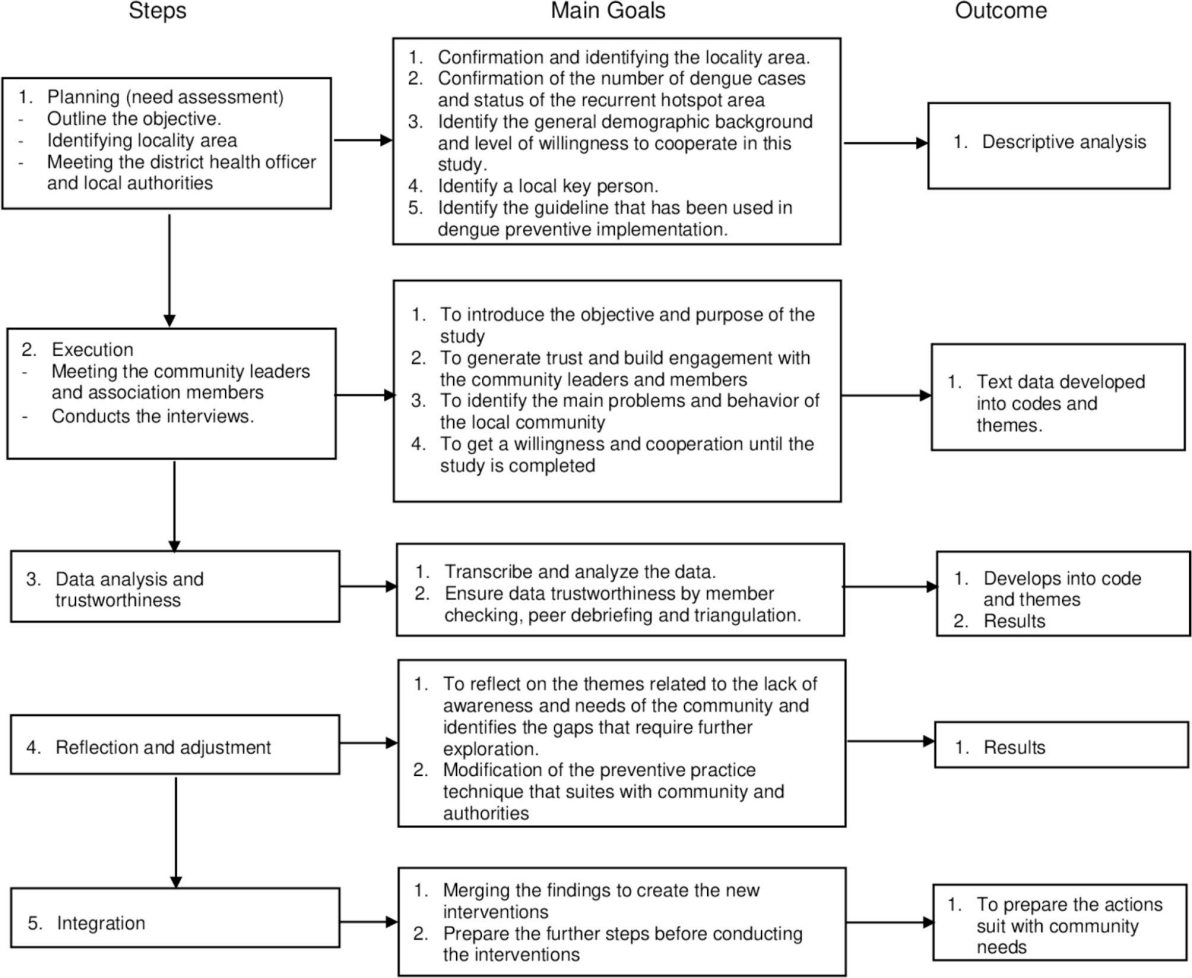
Flowchart of the systematic steps completed during the study

Data collection was conducted using an iterative process. Figure 1 shows the steps of data collection in this study (Fig. 1).

#### Step 1. Planning (need assessment)

Secondary data from the iDengue website and the Selangor State Health Department portal were reviewed in the first stage of study planning to understand the epidemiology of dengue cases in the area. Following that, a series of meetings with representatives from the district health office and local authorities were organized to convey the study objectives before obtaining their permission to conduct the study. During this phase, the foundation of CBPR was applied. Authorities’ opinions were sought on the most suitable locality to conduct the study. Guidelines on dengue control and eradication were also requested from the stakeholders.

#### Step 2. Execution

The successful implementation of this study requires the proactive involvement of the community and stakeholders. Thus, the study team must first evaluate the community’s requirements and apprehensions. To achieve this objective, a set of interviews was held with prominent individuals in the community, including community leaders, religious leaders, health officers, and local authorities to obtain information about localities that had been repeatedly classified as hotspot areas.

Furthermore, qualitative interviews were performed with selected participants via IDIs and discussions through FGDs to gain a deeper understanding and valuable insight from the perspective of the stakeholders and community. Convenience sampling was applied to recruit individuals who were readily available and willing to share their experiences and opinions, thus allowing the research team to explore diverse viewpoints and capture a wider range of perspectives. Data were collected until data saturation was reached, i.e. when no new information emerged from the interviews with the participants. The IDIs and FGDs spanned from 60 to 90 min. All interviews were audio-recorded.

#### Step 3. Data analysis and trustworthiness

In this study, ATLAS. Ti Windows (version 9.1.7.0) was used for qualitative data collection and analysis. In Steps 1, 2, and 3, all interviews were conducted in the Malay language. C.S.S., Z.I.Z., and H.O. who are fluent in English translated the quotes from Malay to English. All field notes and interview transcripts were transferred to a computer and stored securely using password-protected devices. Field notes were taken to ensure transferability and quotes from interview transcripts were used to generate confirmability and dependability.

The study findings were guided by Braun and Clark’s reflexive thematic analysis [26]. Employing a deductive-inductive approach for coding, patterns and meanings were identified, compared, and interpreted across datasets [26]. N.A.S. transcribed the interviews and then iteratively read the data in depth to familiarize herself with the dataset. From the transcripts, N.A.S. created codes and themes. Additionally, any emerging codes and themes from iterative focus group discussions were deliberated to prevent misinterpretation. To ensure the richness of the information contained in the raw dataset, C.S.S. and Z.I.Z. facilitated the generation of codes and themes. As part of the final review, H.O. and the coders agreed on the codes and themes that were generated inductively based on the researchers’ reflexivity. Coauthors were additional coders to increase the data collection [26]. Adding full descriptions to data can enhance its transferability and rigor and facilitate contextual evaluation. Furthermore, the data were recorded, and a systematic analysis was conducted using a coding system. Member checking was conducted following each step to ensure data rigor and credibility. The member checking ensured that the data were trustworthy and reduced researcher bias after the interviews. This can minimize misinterpretation of facial expressions during interviews. Finally, the study participants were asked to verify the accuracy of the data following a discussion of the findings.

#### Step 4. Reflection and adjustment

Understanding the needs of the community and the gaps in current practice were the crucial aims of Step 4. The researchers must consider the different preventive practices that can be adapted to best fit the needs of the community and the authorities by evaluating the guidelines and reflecting on the interview findings.

Changing communication methods to enhance the outcomes is vital. Thus, it is a commonly applied intervention method. For example, the CBPR method can be taken to encourage the community to be more enthusiastic about the dengue prevention and control program to reduce the number of recurrent dengue hotspot areas. By spending time reflecting on the community’s needs and adjusting current practices based on their potential concerns, the right actions that benefit both parties can be taken.

#### Step 5. Integration

In the final steps, the study findings are integrated to generate new interventions. Researchers should take into account all the study findings and observations to combine all of them into one intervention program. This integrated understanding then serves as the basis for developing new approaches and policies for addressing community needs. This process requires careful analysis and consideration of the findings to ensure that the new interventions will be comprehensive, effective, and most importantly, tailored to community needs. Finally, it is imperative to evaluate the new interventions to ensure that they achieve the desired outcomes.

### Patient and public involvement

No patients were involved in the planning or conduct of this study. However, some members of the public were recruited as study participants for the qualitative interviews.

## Results

### Demographic characteristics of participants

**Fig. 2:**
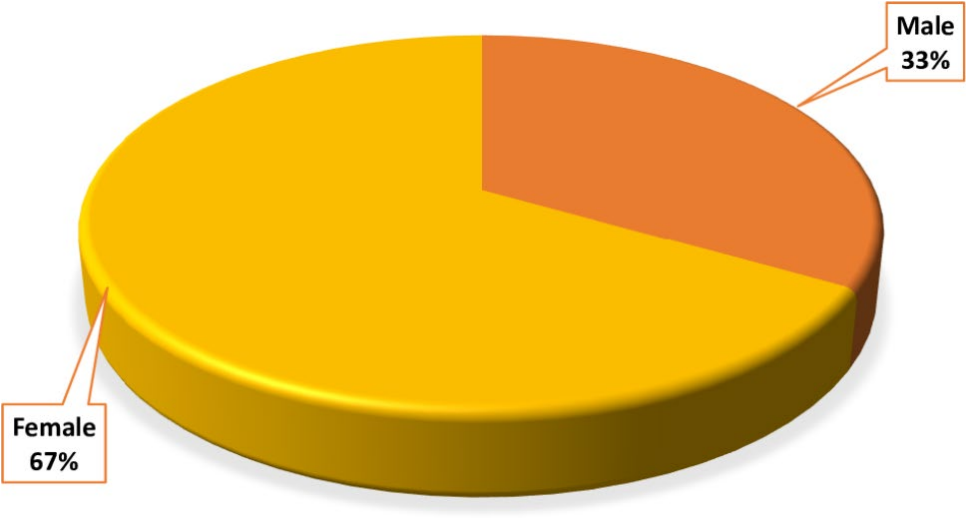
Pie chart showing the distribution of participants by gender

This section provides a graphical representation of demographic characteristics of the participants, focusing on age and gender. Figure 2 illustrated the distribution of participants based on gender, with 33% males and 67% females.

**Fig. 3:**
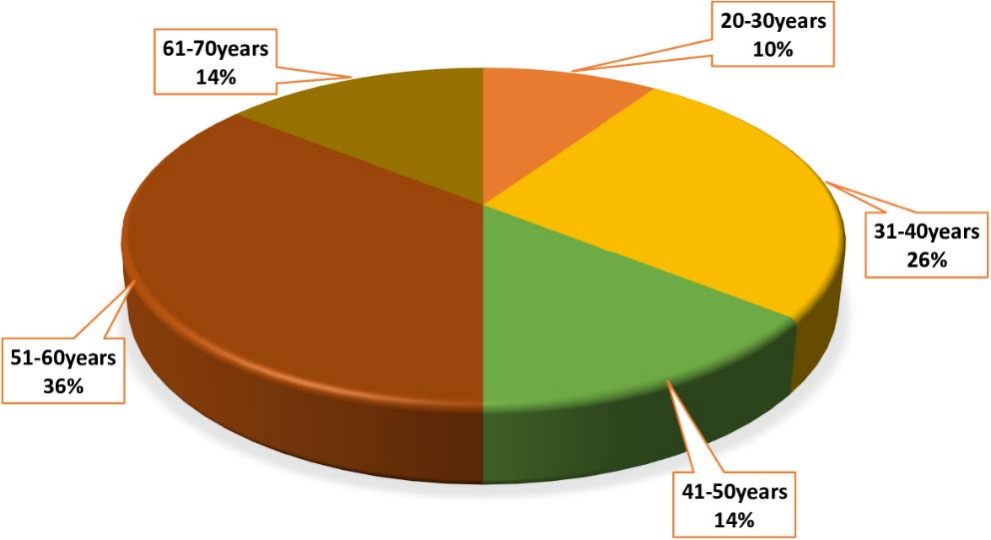
Pie chart showing the distribution of participants by age

Meanwhile, Fig. 3 presents the distribution of participants by age group. It is evident that the modal age group is 20–30 years (4 participants, 10%), followed by the age groups 31–40 years (11 participants, 26%), 41–50 years (6 participants, 14%), 51–60 years (15 participants, 36%) and 61–70 years (6 participants, 14%), respectively.

### Community needs in Aedes mosquito and dengue fever prevention

#### Theme 1: appropriate and firm actions by the authorities

The interviewees agreed on the necessity for authorities to take decisive and effective measures to mitigate the impact of dengue outbreaks on local residents. Nonetheless, jurisdiction disparity often deters the executive of these measures. For example, under the guidelines implemented by the district health office, a notice to compound the residence owner may be promptly issued upon discovering a breeding site during an inspection. However, only a caution will be issued by municipal officers as a reprimand in the same situation.

When I complained about this neighbor’s messy place, the authorities inspected the area and found 31 mosquito breeding bottles. However, what they do is just give advice and leave. In the end, it’s us, the surrounding neighbors who will suffer from dengue fever. [FGD P14]

If we can have the authorities, such as the district health office, to issue fine notices, the community would be more cautious. We may not be able to issue penalties as the existing laws are challenging to enforce. [IDI LA06]

Most of the local community members responded that only fines or compounds would increase individual awareness about their surrounding areas. It would also serve as a warning to other residents to be more attentive in cleaning their premises to prevent the formation of Aedes breeding sites. Additionally, they stressed that issues related to environmental cleanliness, such as blocked sewers and uncollected waste should receive greater attention. Often, they noticed a delayed response when submitting the relevant complaints to the authorities. They wished for timely action on the issue of environmental cleanliness to reduce the number of breeding sites.

This community only takes it seriously when they receive fines. They only pay attention when warnings are given but they do not act until fines are issued. [IDI CL12]

They (the community) are not afraid of dengue because they have not been affected personally. That is why they are not of concern. Community cooperation efforts no longer work. It would be better to issue fines as it is a more straightforward solution. [FGD P07,P20]

#### Theme 2: multisectoral partnership for integrated dengue control (Participatory Research)

Multisectoral partnerships or participatory research design are needed to generate a sustainable dengue control program. In this study, the participants expressed a strong desire to be actively engaged in any efforts against dengue fever. Their local knowledge, experiences, and perspectives will be valuable in the development of appropriate and context-specific solutions. They also highlighted that a participatory approach would empower the community, promote ownership, and foster a sense of responsibility in combating Aedes mosquitoes. In addition, when a multisectoral partnership is used, residents are more enthusiastic and proactive in dealing with the Aedes mosquito breeding site.

We have previously organized a community clean-up involving multiple parties. Many agencies were setting up booths at that time, and even the fire department and police were called in. It was a lively atmosphere and many residents participated enthusiastically. [FGD P10]

We need assistance from various parties, including universities, to reach the community. We are unable to carry out comprehensive efforts with limited staff. [IDI LA08]

One aspect highlighted by the stakeholders was the need for community-based participatory of initiatives and mobilization. Under this concept, the community should be made aware of the importance of organizing awareness campaigns, clean-up drives, and health education. By involving residents, universities, religious institutions, and local organizations in these initiatives, it is possible to improve awareness and promote behavior change about dengue prevention. However, to ensure success, these community-driven efforts should be supported with sufficient funding, technical assistance, and capacity building.

After the university conducted a program here and distributed survey forms, it has benefited me greatly. When I read the questions, it made me think and search for answers to those questions. The questions indirectly added to my knowledge. [IDI FL13]

At the local authority level, we will utilize any available resources for health promotion campaigns on dengue. Currently, we can only use the existing signboards. We would greatly appreciate it if the university could assist us in providing materials and larger signboards. [IDI LA06]

#### Theme 3: motivation through incentives and rewards

To ensure more comprehensive community participation, tangible incentives and rewards should be offered as they have been linked with better community engagement and participation in mosquito control efforts. Some of the suggestions put forward in the interviews included the provision of financial incentives for implementing mosquito control. For example, every time a “gotong-royong” is held, a potluck or shared costs in meal preparation should be included to attract more participation from residents. Such incentives represent a token of appreciation toward the efforts of community members and reduce any additional costs associated with mosquito control.

To encourage others to participate in the community clean-up program, we need to provide incentives such as breakfast. If free food is available, more people would be motivated to join the clean-up efforts. [IDI CL12]

Perhaps for future programs, we can request the council members or health office to allocate funds for providing breakfast. That would be the only feasible option to arrange breakfast during the program. [FGD P01, P16]

The interviewees also highlighted the potential of using nonmonetary incentives to foster community engagement. They suggested organizing competitions or events that reward communities or neighborhoods with the lowest mosquito breeding indices. The incentives can be in the form of certificates, vouchers, or hampers. Such rewards can create a sense of achievement and pride, thus promoting unity within the community.

Perhaps we can organize a “seek and find” activity in each zone. The cleanest zone can be rewarded with prizes or incentives. This would create a sense of excitement and motivation among the community members. [IDI P14]

The community needs some form of reward for their efforts. When organizing programs, it would be beneficial to provide incentives. We can consider offering vouchers or other rewards to encourage participation and as a recognition of their contributions. [FGD P15]

#### Theme 4: sustaining community engagement and education

The interviewees acknowledged that community engagement and education should be long-term comprehensive programs rather than short-term sporadic campaigns. Festivals or community events dedicated to dengue prevention would serve as engaging and interactive platforms for raising awareness and educating the community. These events should be organized periodically, such as annually or biannually, to ensure ongoing community engagement. Furthermore, continuous education is vital to equip the community with the latest knowledge and skills to combat Aedes mosquitoes and dengue fever.

Indeed, when organizing large-scale programs, more people are likely to participate and information can be easily disseminated. It may be beneficial to hold such events occasionally and increase the number of exhibition booths for residents. This will provide a platform for effective communication and engagement with the community. [IDI HA02]

In addition, the interviewees emphasized the importance of involving young people, especially teenagers in dengue prevention efforts. They recognized that young individuals play a significant role in shaping community behaviors and influencing their peers and family members. Furthermore, it was noted that house of worship also contributes to community education efforts, highlighting the necessity for a sustained reminder of preventive measures.

In the future, my suggestion is to engage a large number of teenagers, especially those between 14 and 18 years old. We should encourage them to join as volunteers and cultivate their interest in combating the Aedes mosquito. [FGD P10]

As the longtime chairman of this mosque, I’ve had health officer visit us before. They came to distribute health pamphlets to the congregation after Friday prayers. It was a great initiative because that’s when many people are present and interested in the materials being distributed. I even arranged for the health officier to give a short health talk during that time. So, using the mosque as a way to reach out to the community is really beneficial and something we wholeheartedly support. [IDI FL13]

## Discussion

To the best of our knowledge, this is the first study of its kind in the region of Hulu Langat, Selangor. This qualitative study aimed to explore the community’s needs in combatting Aedes mosquitoes and dengue fever in a recurrent hotspot area based on the ideation model of communication. Effective interventions can be designed by gaining a deeper understanding of the factors influencing the community’s behavior and attitudes toward Aedes mosquitoes [27]. One crucial aspect highlighted by the stakeholders was the importance of identifying the underlying needs and concerns of the community. The study findings shed light on the community’s perspectives, concerns, and needs regarding the prevention and control of Aedes mosquitoes as well as the transmission of dengue fever.

Human behaviors are complex as they are influenced by multiple factors, including social norms, attitudes, and social constructs [19, 28]. Thus, it is vital to understand the community beliefs and perspectives of the disease, as well as the challenges faced so that appropriate and sustainable disease control interventions can be developed and implemented [29]. The interventions must be customized to the needs of the local populations to ensure acceptance and adherence [30, 31]. Additionally, interventions should be flexible to adapt to any changes in the local environment and communities [31, 32]. Our findings reiterated these important features that should be emphasized when designing and implementing community-based interventions. Furthermore, our study also highlighted the need for further research and development to improve the effectiveness and sustainability of interventions in different cultural contexts.

In today’s world, rallying community involvement can be challenging compared to a few decades ago [33]. There is a decrease in the spirit and unity within a neighborhood, especially regarding volunteering in community activities [34]. Busy lifestyles consisting of work schedules on weekdays and family commitments on weekends are the main reasons behind poor community engagement. Furthermore, some neighborhoods prioritize their interests over the collective interest of the whole community, thus creating more challenges in combatting Aedes mosquitoes [35, 36]. To date, all the published literature strongly advocates the need for close involvement of local communities backed by solid policies, strong alliances, and capable leaders to ensure active community participation in dengue prevention initiatives [37]. Thus, our study contributed to the literature by reporting how the local community can be motivated to participate in activities or socialize with its neighbors.

In this study, one prevalent theme that emerged was how many deemed fines or penalties to be an effective mechanism in the prevention and control of dengue. The participants argued that fines act as a deterrent to discourage negative behaviors that contribute to the spread of the disease. However, they agreed that financial punishment might have only limited deterrent effects [38]. In some instances, certain individuals may be more inclined to engage in illegal activities if the financial penalty is the only consequence that they will face [39]. Therefore, while fines can help to enforce certain community standards, their effectiveness is not absolute. Continuous investment in public education campaigns is crucial to raise awareness and promote voluntary compliance [40, 41]. Additionally, clear national guidelines must be established to reduce disparities between different jurisdictions so that local government officials can enforce regulations more effectively [42].

The role of fines and penalties as deterrents in dengue prevention demands a nuanced analysis from a behavioral economics perspective [43]. Beyond punitive measures, cognitive biases and heuristics can significantly influence the effectiveness of such interventions [43]. Public opinion and perceptions of fairness become paramount, emphasizing the necessity for a comprehensive approach that considers the psychological impact of punitive measures. The importance of collaboration with the community extends beyond surface-level acknowledgment, urging a deeper examination of power dynamics within collaborative efforts. Identifying and addressing power imbalances is critical for ensuring equitable participation among diverse stakeholders [44]. Trust-building mechanisms emerge as a central theme, underscoring the need for strategies to establish and maintain trust for sustainable community engagement [44].

This study reinforces the crucial role of collaboration with the community. Multisectoral partnerships such as participatory research have been shown to be effective in raising awareness and rallying local communities in dengue control efforts [45, 46]. By engaging relevant stakeholders such as community members, healthcare professionals, and government agencies through PR, dengue control strategies can be more effective [47, 48]. All these partnerships can foster close collaboration and knowledge sharing that lead to a more comprehensive and community-centered approach to combating dengue [49, 50]. The PAR approach also recognizes the valuable knowledge and expertise possessed by community members who have first-hand experience and understanding of the local context. Often, the success and sustainability of disease prevention programs increase when the local community actively participates in the development and implementation of control techniques [51].

Building on the foundation of participatory research, it becomes evident that sustained community collaboration offers a dynamic platform for continuous improvement in dengue control [52]. Long-term engagement establishes a feedback loop, enabling the adaptation of strategies based on real-time insights from the community [52, 53]. Recommendations include the implementation of targeted educational campaigns, community-led clean-up initiatives, and the establishment of local task forces dedicated to mosquito control. The health authorities should regularly update the community, academic partners, and stakeholders on disease updates, vector behavior and other pertinent epidemiological information to the local community.

Furthermore, the local residents underlined the importance of incentives, be it in the form of financial rewards or free meals in encouraging greater community participation. Similar findings have been reported in previous studies whereby incentives and rewards can motivate individuals to participate in community engagement and public health programs [50, 54]. However, financial constraints at the authority level may limit the ability of local officials to provide financial incentives to community members. In such cases, alternative approaches can be explored to address the community’s needs by using the available resources or soliciting alternative support. Based on the PR concept, one possible approach is to seek partnerships or sponsorship as a source of financial support for community initiatives.

In addition to financial incentives, non-monetary rewards can also be an important source of motivation for community members. For example, recognition programs, certificates of appreciation, or public acknowledgments of individuals’ or groups’ contributions to dengue prevention efforts can be a sign of recognition. Authorities can also consider the designation of exemplary community participation in dengue prevention as a model community group. By showcasing their success to others, these recognitions validate their efforts and serve as a source of pride and motivation for community members [34]. More importantly, the achievements and best practices of one community group can be used by the authorities to inspire other neighborhoods to follow suit in their dengue prevention initiatives.

Finally, another theme that emerged was the importance of sustaining community engagement and communication. Both stakeholders and community members recognized the significance of providing ongoing education to the community members. However, this depends heavily on adequate financial resources. In line with the recommendations from a study in Burkina Faso [35, 55], public health authorities should be constantly educating and communicating with the public, not just during outbreaks. They should prioritize sustainable sustained efforts as an integral part of their long-term strategy in health promotion and disease prevention. The fundamental way to achieve this is by securing consistent funding and allocating a dedicated budget for health education and communication. They must ensure the availability of resources, materials, and qualified personnel to effectively engage and educate the community. More importantly, this budget should be sufficient to cover not only the immediate needs during outbreaks but also to sustain efforts in ensuring long-term behavioral change and disease prevention.

To ensure sustained community involvement in disease prevention programs, authorities must prioritize long-term strategies over sporadic campaigns [55]. Active involvement of young people in community engagement and education initiatives is a practical approach. Youths play a crucial role in dengue prevention efforts as they can catalyze changes in their communities. An example from Indonesia showcased a successful community-based dengue prevention intervention that involved young people through education and cleaning up mosquito breeding sites in and around schools [56–58].

Leveraging technology and social media platforms is an effective way of reaching and engaging young individuals. Informative and interactive online campaigns, mobile applications for dengue prevention, and engagement with social media influencers are a few ways to facilitate information dissemination and raise awareness among young people in today’s digital world. In the long term, targeting young individuals as the focal point of change and encouraging them to assume leadership roles in dengue prevention initiatives can empower them and leverage their fresh perspectives to drive meaningful change in the fight against the dengue epidemic.

In conclusion, it is essential to place strong emphasis on the need for community education regarding the severity of dengue fever. This disease poses a significant threat to public health, with potentially fatal outcomes. By educating the community about the serious nature of dengue and its potential for high mortality rates, we empower individuals to recognize the gravity of the situation. Through understanding the risks associated with dengue, as proposed by the Health Belief Model theory [59, 60], community members are more likely to take proactive measures to protect themselves and their loved ones from this dangerous illness. Therefore, it’s paramount that we prioritize comprehensive education initiatives to ensure that everyone comprehends the seriousness of the dengue threat.

### Limitation

While this study contributes valuable insights into understanding community needs in combating Aedes mosquitoes and dengue fever within urban hotspot areas, it is important to acknowledge certain limitations that may affect the interpretation and generalizability of the findings. Firstly, the evaluation of control activities, such as fogging, in reducing dengue transmission within the urban community, was not explicitly addressed in this study. However, the importance of assessing the effectiveness of such interventions, including fogging during outbreaks, to understand their impact on dengue incidence rates is recognized. Additionally, the potential for community participatory approaches in evaluating the effectiveness of control activities, including the use of mobile apps to alert on breeding areas and assess the impact of interventions, represents another area for future research. While not within the scope of this study, incorporating community participatory methods could enhance the effectiveness of interventions and empower communities to take proactive measures in dengue prevention. These limitations highlight areas for further research and underscore the need for a comprehensive approach to addressing dengue fever in urban communities.

## Conclusion

This study provided important insights regarding the community’s needs in combating Aedes mosquitoes and dengue fever. A multifaceted approach involving various stakeholders such as local authorities, universities, faith leaders or religious local leaders and residents themselves is vital in addressing the community’s needs. Our findings emphasize the need to raise awareness, provide practical resources, and foster a sense of responsibility and ownership among community members to create effective and sustainable strategies against dengue fever. Their involvement can serve as a powerful catalyst in continuously reminding the community to take proactive measures against Aedes mosquitoes and dengue fever. Furthermore, the active involvement of teenagers as volunteers can nurture their interests in this cause, making them a valuable asset in the fight against Aedes mosquitoes. Overall, our study highlights the significance of collaboration, resource allocation, and community engagement to effectively tackle the challenges of dengue fever with the ultimate aim of achieving a healthier and safer environment for all.

### Electronic supplementary material

Below is the link to the electronic supplementary material.

Supplementary Material 1

## Data Availability

Data are available on reasonable request. These data are available from the corresponding author (hida@ukm.edu.my) upon reasonable request.

## Abbreviations

LO: local officer
DO: district health officer
CBPR: Community- based participatory research
FL: faith leader

## Declarations

### Patient and public involvement

Neither patients nor the public were involved in the design, conduct, reporting, or dissemination plans of this research.
