# Supplementary material for: Exploring community needs in combating aedes mosquitoes and dengue fever: a study with urban community in the recurrent hotspot area
Source: BMC Public Health. 2024 Jun 20;24:1651. doi: 10.1186/s12889-024-18965-1 (PMC11191142; doi:10.1186/s12889-024-18965-1)
Supplement: Supplementary file 1 — Supplementary Material 1 [file 12889_2024_18965_MOESM1_ESM.docx]

Supplementary File: The themes and quotes from participants

| **Themes** | **The quotes from participants** |
| --- | --- |
| Appropriate and firm actions by the authorities | *This community only takes it seriously when they receive fines. They only pay attention when warnings are given but they do not act until fines are issued.* [IDI CL12]  *They (the community) are not afraid of dengue because they have not been affected personally. That is why they are not of concern. Community cooperation efforts no longer work. It would be better to issue fines as it is a more straightforward solution.* [FGD P07, P20]  *When we give advice, they don't take any action. But when we start issuing fines to the neighbors, they begin to take action.* [IDI LO 07]  *Most communities here are preoccupied with their own affairs. Even if there are no issues at hand, they won’t pay attention to the environmental conditions-whatever happens, they remain indifferent. Only when there’s summons, then they start to take notice* [FGD P13] |
| Multisectoral partnership for integrated dengue control (Participatory Action Research) | *We have previously organized a community clean-up involving multiple parties. Many agencies were setting up booths at that time, and even the fire department and police were called in. It was a lively atmosphere and many residents participated enthusiastically.* [FGD P10]  *We need assistance from various parties, including universities, to reach the community. We are unable to carry out comprehensive efforts with limited staff* [IDI LA08]  *After the university conducted a program here and distributed survey forms, it has benefited me greatly. When I read the questions, it made me think and search for answers to those questions. The questions indirectly added to my knowledge.* [IDI FL13]  *At the local authority level, we will utilize any available resources for health promotion campaigns on dengue. Currently, we can only use the existing signboards. We would greatly appreciate it if the university could assist us in providing materials and larger signboards. [IDI LA06]* |
| Motivation through incentives and rewards | *To encourage others to participate in the community clean-up program, we need to provide incentives such as breakfast. If free food is available, more people would be motivated to join the clean-up efforts.* [IDI CL12]  *Perhaps for future programs, we can request the council members or health office to allocate funds for providing breakfast. That would be the only feasible option to arrange breakfast during the program*. [FGD P01, P16]  *Perhaps we can organize a "seek and find" activity in each zone. The cleanest zone can be rewarded with prizes or incentives. This would create a sense of excitement and motivation among the community members*. [IDI P14]  *The community needs some form of reward for their efforts. When organizing programs, it would be beneficial to provide incentives. We can consider offering vouchers or other rewards to encourage participation and as a recognition of their contributions.* [FGD P15] |
| Sustaining community engagement and education | *Indeed, when organizing large-scale programs, more people are likely to participate and information can be easily disseminated. It may be beneficial to hold such events occasionally and increase the number of exhibition booths for residents. This will provide a platform for effective communication and engagement with the community.* [IDI HA02]  *In the future, my suggestion is to engage a large number of teenagers, especially those between 14 and 18 years old. We should encourage them to join as volunteers and cultivate their interest in combating the Aedes mosquito*. [FGD P10] |
